# Longer daily oxygen use associates with more adverse events, symptoms, and worse health status in long-term oxygen therapy

**DOI:** 10.1177/14799731251366962

**Published:** 2025-08-08

**Authors:** Filip Björklund, Magnus Ekström

**Affiliations:** 1 Faculty of Medicine, Department of Clinical Sciences Lund, Respiratory Medicine, Allergology, and Palliative Medicine, RinggoldID:59568 Lund University, Lund, Sweden

**Keywords:** long-term oxygen therapy, duration, adverse events, adherence, health status, sleep

## Abstract

**Introduction:**

Use of long-term oxygen therapy (LTOT) for more than 15 h per day does not reduce mortality or hospitalizations, but may increase the risk of adverse events. We evaluated the relationship between daily oxygen use duration and adverse events, symptoms, and health status in patients on LTOT.

**Methods:**

This was a cross-sectional survey study of a random sample (*N* = 650) of adults with ongoing LTOT in Sweden. Oxygen use (h/day) was reported, and associations were analyzed with adverse events, symptom severities (revised Edmonton Symptom Assessment System), sleep duration and quality, and health status (COPD assessment test [CAT]).

**Results:**

In total, surveys from 204 patients were analyzed; 60% female, mean age 75.3 (SD 8.7) years. Swedevox baseline characteristics were similar between sampled respondents and non-respondents. Patients reporting 24 h of daily oxygen use (53.4%) also reported a higher number of total adverse events, higher ratings of dyspnea, depression and anxiety, and worse health status, compared to those reporting fewer hours of oxygen use. A longer daily duration of oxygen use also associated with a higher number of experienced adverse events, higher ratings of dyspnea and anxiety, and worse rated health status in crude and adjusted linear regression models. No associations were seen between oxygen use duration and sleep quality or duration.

**Conclusion:**

More adverse events, a higher severity of some symptoms, and worse health status are seen among patients with a longer daily duration of oxygen use. Further research is needed to establish evidence of causality.

## Introduction

Long-term oxygen therapy (LTOT), prescribed for 15 h per day or longer, improves survival in persons with chronic obstructive pulmonary disease (COPD) and chronic severe resting hypoxemia,^1,2^ with additional observational data further supporting a decrease in the number of exacerbations and hospitalizations after initiation of LTOT.^3^ In clinical practice, LTOT is commonly also prescribed for patients with hypoxemia due to other conditions than COPD, such as interstitial lung disease (ILD), although evidence is lacking.^4,5^

The optimal daily duration of oxygen use for patients with LTOT is not fully known. While most current guidelines, based on data from the original trials of LTOT,^1,2^ recommend prescription of LTOT for at least 15 h per day, the choice of whether to recommend further daily usage up to 24 h has previously been informed only by comparative interpretations of the original studies.^6^ The recent REDOX trial^7^ showed no decrease in the risk of hospitalization or death at 1 year for patients prescribed continuous 24-h LTOT, compared to those prescribed LTOT for 15 h per day, and no between-group differences were seen for self-reported breathlessness or health status. Data on patient-reported outcomes in REDOX were however limited with respect to sample size, and other consequences of the selected daily oxygen usage time, such as the effect on symptoms and adverse events, are incompletely explored.

Patients in chronic respiratory failure who initiate LTOT generally experience a large burden of symptoms, but there is little evidence to support whether these improve or worsen with LTOT usage.^8–13^ On one hand, use of LTOT has in some nonrandomized studies been suggested to positively influence quality of life factors,^14,15^ while on the other hand poor adherence is regularly found in real-world studies of LTOT users,^9,16–19^ who also commonly report potentially treatment-related adverse effects and symptoms, such as localized irritation, limitations in mobility and social stigmatization.^20,21^ We hypothesized that a longer daily oxygen usage time would not associate with an improved quality of life, but would associate with a higher risk of adverse events and symptoms.

The aim of this study was to evaluate the associations between longer daily oxygen use duration and adverse events, symptom severities, and health status in patients on LTOT.

## Methods

### Study design and population

This was a cross-sectional survey study of 650 patients aged ≥18 years, randomly selected from patients with ongoing LTOT in the National Registry for Respiratory Failure (Swedevox) registry on January 12^th^, 2021, with surveys collected between March 9^th^ and April 18^th^, 2021. Swedevox prospectively covers around 85% of all patients with ongoing LTOT in Sweden, who on the selection date numbered 2,327.^22^ Swedevox has previously been validated against medical records for key variables.^23^ The survey database has previously been used to evaluate adverse events in relation to health status among LTOT users.^20^

Each sampled patient received a postal survey, and was asked to return it in an enclosed envelope after completion. Patients who did not return their survey, and did not otherwise contact the researchers regarding their participation within 2 weeks, were sent a postal reminder. Patients who did not resend their questionnaire after obtaining a reminder, or who resent their questionnaire without providing information on daily time of oxygen use, were in this study classified as non-respondents.

Ethical approval for the study was granted by the Swedish Ethical Review Agency (Identifier 2020-04528). Patients were informed that by returning the survey they consented to participating in the study. The study is reported in accordance with the Strengthening the Reporting of Observational Studies in Epidemiology (STROBE) guidelines.^24^

### Assessments

From Swedevox, baseline data from the time of LTOT initiation were obtained regarding age, sex, underlying cause for LTOT initiation, blood gas analyses, and WHO performance status.

In the postal questionnaire, patients were asked to report their height and weight, daily oxygen prescription time, actual daily oxygen use duration, and smoking history. For adverse events, patients were asked to report the frequency of occurrence (never, yearly, multiple times per year, monthly, weekly, or daily) for 24 pre-specified events listed below. Symptom severity was assessed using the revised Edmonton Symptom Assessment System (ESAS-r), which measures the severity of nine prespecified symptoms common in end-of-life care on 11-degree ordinal rating scales. The ESAS-r is commonly used for symptom screening and follow-up in research and clinical care, and is available in a validated Swedish translation.^25,26^ Sleep duration and sleep quality were separately assessed, the later using a five-degree ordinal rating scale (very good, good, fairly good, poor, or very poor). Health status was assessed using the COPD Assessment Test (CAT) total score, which has previously been validated as a measure of health status and HrQoL among patients with both COPD and ILD, who together make up the vast majority of patients on LTOT in Sweden.^27,28^ The recall period for all instruments used in this study was specified as the last 2 weeks before survey completion.

### Statistical analyses

Swedevox baseline data available for all sampled patients was compared between survey respondents and non-respondents to assess potential selection bias. No data were imputed. For adverse events, prevalent adverse events were defined as those reported to occur monthly or more often. For each patient, the number of prevalent adverse effects was summed by four categories: Local (including dry mouth, nasal congestion or drip, dry nose, hoarseness, nasal bleeding, pain or soreness in nose, worsened or changed sense of smell, worsened or changes sense of taste, irritated skin or chafing, pain or soreness in throat, dental issues, and pain or soreness in mouth), Systemic (including increased tiredness, increased amount of phlegm, increased thirst, cough, difficulty sleeping, dizziness, reduced appetite, morning headache, and headache during other parts of the day), Practical (including reduced mobility or physical activity, trip or fall, and burn injury or fire), and Social (including sense of loneliness or social isolation, and sense of shame), as well as in a total sum. To avoid a compound effect of missing values upon summing, adverse effects without an indicated frequency were categorized as non-prevalent.

Baseline characteristics and survey outcome data were tabulated by the daily oxygen use duration self-reported in the questionnaire (categorized as 24 h/day vs <24 h per day, as this resulted in an approximately even distribution, and associations with adverse events might be highest for continuous oxygen use). As descriptive statistics, means with standard deviations (SD) and medians with interquartile ranges (IQR, 25^th^-75^th^ percentile) were used for continuous normally and non-normally distributed data respectively. Fisher’s exact tests and Wilcoxon rank-sum tests were used to compare the distributions of outcome variables between the groups. The frequency of patients reporting oxygen use below or above their prescription, and the median and distribution of differences from prescription for these patients was calculated.

Associations between daily oxygen use (in hours per day) and the total number of reported adverse events, symptom severities, and health status were analyzed using linear regression. In the regression analyses, total numbers of adverse events, sleep quality ratings, and CAT scores were normalized by division to 11-point scales, in order to enable comparison with the 11-degree symptom severity scales of the ESAS-r. Analyses were performed crude and adjusted for potentially confounding factors (age, sex, underlying cause for LTOT, arterial partial pressure of oxygen and carbon dioxide (PaO2 and PaCO2) on air and oxygen, baseline World Health Organization (WHO) performance status, and smoking history).

Statistical analyses were performed using Stata version 17 (StataCorp LP; College Station, TX).

## Results

In total, 204 surveys (31.4%) were returned with provided information on daily oxygen use, and were included in analyses. The 204 respondents were majority female (60.3%), with a mean age of 75.3 (SD 8.7) years. A majority of respondents were ever-smokers, with COPD as the main underlying cause for LTOT initiation (table 1). Baseline characteristics registered in Swedevox were similar between respondents and non-respondents (Supplemental table S1).

**Table 1.** Baseline characteristics by reported daily oxygen duration.

	Self-reported daily oxygen duration	
	24 h/day *n* = 109	<24 h/day *n* = 95
Age at survey (years), mean (SD)	75.1 (8.8)	75.6 (8.6)
Months since starting LTOT, median (IQR)	31 (14–49)	16 (7.0–34)
Female	70 (64.2%)	53 (55.8%)
BMI (mean, SD)	26.8 (7.1)	26.9 (6.9)
Main underlying cause for LTOT		
COPD	77 (70.6%)	76 (80.0%)
ILD	16 (14.7%)	7 (7.4%)
Other	15 (13.8%)	12 (12.6%)
Missing	1 (0.9%)	0 (0.0%)
Baseline PaO_2_ on air, kPa (mean, SD)	6.5 (0.85)	6.7 (0.62)
Baseline PaO_2_ on oxygen, kPa (mean, SD)	8.7 (1.0)	8.8 (1.2)
Baseline PaCO_2_ on air, kPa (mean, SD)	5.9 (1.5)	5.6 (1.2)
Baseline PaCO_2_ on oxygen, kPa (mean, SD)	6.0 (1.1)	5.8 (1.1)
Baseline FEV1, L (median, IQR)	1.0 (0.7–1.5)	1.1 (0.7–1.6)
Baseline FEV1, % predicted (median, IQR)	42.2 (31.4–57.8)	44.6 (32.1–58.4)
Ever-smoker	91 (84.3%)	74 (84.1%)
WHO performance status		
1	9 (8.3%)	14 (14.7%)
2	51 (46.8%)	41 (43.2%)
3	21 (19.3%)	19 (20.0%)
4	10 (9.2%)	6 (6.3%)
Missing	18 (16.5%)	15 (5.8%)

BMI: Body mass index, COPD: Chronic obstructive pulmonary disease, FEV1: Forced expiratory volume in one second, ILD: Interstitial lung disease, IQR: Interquartile range, LTOT: Long-term oxygen therapy, PaCO_2_: Partial pressure of carbon dioxide in arterial blood, PaO_2_: Partial pressure of oxygen in arterial blood, SD: Standard deviation, WHO: World Health Organization.

Among all respondents, 109 (53.4%) reported a daily oxygen duration of 24 h per day (median 24 h [IQR 16-24h]; mean 19.8 h [SD 5.8h]). Oxygen usage time below prescription was reported by 29 patients (14.2%), with a median difference of 4 (IQR 2-7) hours from prescription. Oxygen usage time above prescription was reported by 31 patients (15.2%), with a median difference of 4 (IQR 1-8) hours, out of which 11 reported 24 h of daily oxygen use.

**Figure 1.**
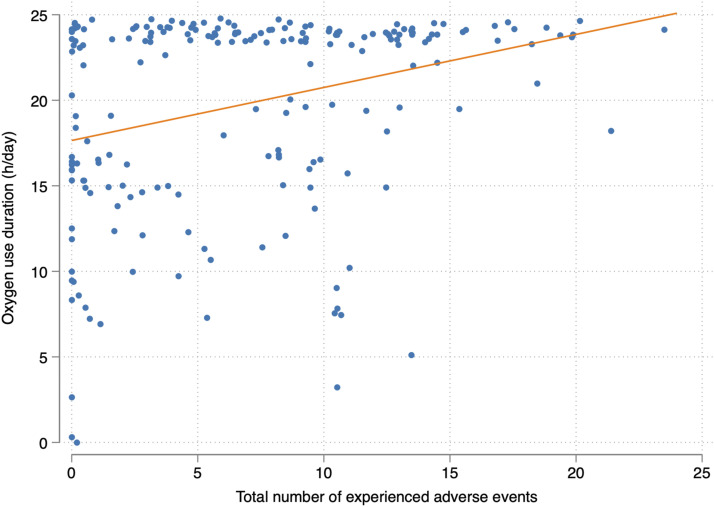
Jittered scatterplot with fitted trend line displaying the relationship between daily oxygen usage time and total number of experienced adverse events.

Respondents reporting a daily oxygen duration of 24 h per day had similar characteristics upon LTOT initiation as those reporting a shorter daily oxygen duration, with respect to age, sex, BMI, underlying cause for treatment, blood gas analyses, smoking history, and performance status (Table 1). The time since starting LTOT tended to be longer among those reporting a daily oxygen duration of 24 h. Patients reporting a daily oxygen duration of 24 h also reported higher numbers of adverse events in the local, systemic, practical, and total groups, significantly higher dyspnea, depression and anxiety ratings on the ESAS-r, and worse health status on the CAT, compared to those reporting oxygen use for fewer hours. There were no significant differences in sleep quality, sleep duration, or other ESAS-r items between the two groups. Relationships between daily oxygen usage time and primary and secondary outcomes are shown in Table 2, Figure 1 and Supplemental figures S1-S3.

**Table 2.** Outcome data, by reported daily oxygen duration.

	Self-reported daily oxygen duration		*p*-value
	24 h/day *n* = 109	<24 h/day *n* = 95	
Number of experienced adverse events by type, median (IQR)			
Local	4 (2–6)	2 (0–5)	<0.001^**^
Systemic	3 (1–5)	1 (0–4)	<0.001^**^
Practical	1 (0–1)	0 (0–1)	0.003^**^
Social	0 (0–1)	0 (0–1)	0.095^**^
Total	8 (4–13)	4 (0–10)	<0.001^**^
ESAS-r score (median, IQR)			
Pain	2 (0-6)	2 (0–5)	0.59^**^
Tiredness	6 (3–8)	5 (3–7)	0.15^**^
Drowsiness	5 (3–7)	3.5 (2–6)	0.068^**^
Nausea	0 (0–1)	0 (0–2)	0.68^**^
Lack of appetite	1.5 (0–5)	1 (0–4)	0.37^**^
Dyspnea	8 (6–9)	7 (3–8)	0.001^**^
Depression	4 (1–8)	2 (1–5)	0.049^**^
Anxiety	4 (0–7)	2 (0–4)	0.008^**^
Overall well-being	5 (3–8)	5 (2–7)	0.12^**^
Sleep duration <6 h/night	13 (11.9%)	15 (15.8%)	0.54^*^
Sleep quality			0.61^*^
Very good	19 (17.4%)	10 (10.5%)	
Good	31 (28.4%)	30 (31.6%)	
Fairly good	42 (38.5%)	40 (42.1%)	
Poor	12 (11.0%)	13 (13.7%)	
Very poor	4 (3.7%)	2 (2.1%)	
Missing	1 (0.9%)	0 (0.0%)	
CAT Score (median, IQR)	25 (21,29)	22 (17,26)	0.001^**^

**Local adverse effects (maximum 12) were defined as**: dry mouth, nasal congestion or drip, dry nose, hoarseness, nasal bleeding, pain or soreness in nose, worsened or changed sense of smell, worsened or changes sense of taste, irritated skin or chafing, pain or soreness in throat, dental issues, pain or soreness in mouth. **Systemic adverse effects (maximum 9) were defined as**: increased tiredness, increased amount of phlegm, increased thirst, cough, difficulty sleeping, dizziness, reduced appetite, headache during other parts of the day, morning headache. **Practical adverse effects (maximum 3) were defined as:** reduced mobility or physical activity, trip or fall, burn injury or fire. **Social adverse effects (maximum 2) were defined as:** sense of loneliness or social isolation, sense of shame.

^*^Fisher’s exact test ^**^Wilcoxon signed-rank test.

CAT: Chronic obstructive pulmonary disease assessment test, ESAS-r: Revised Edmonton symptom assessment system, IQR: Interquartile range.

**Figure 2.**
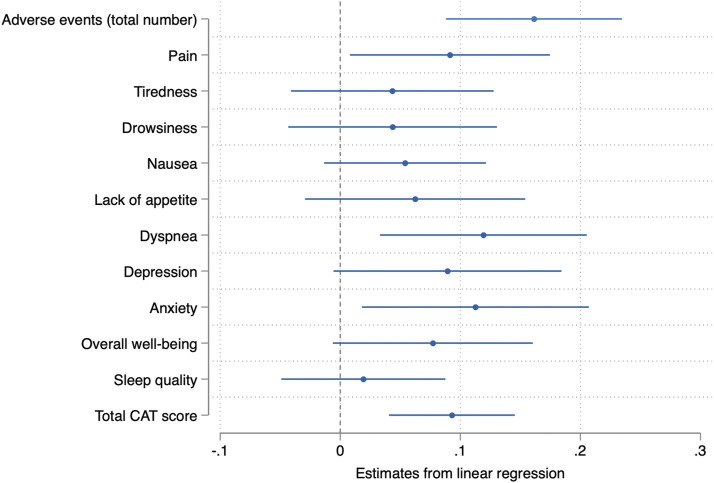
Associations between daily oxygen use duration and adverse events, severity of symptoms, and health status. Symbols represent estimates from adjusted linear regression, with 95% confidence intervals. Coefficients represent the mean shift in outcome variable per additional 1 hour of daily oxygen duration. A positive estimate thus reflects an increase in the outcome variable (i.e. higher number of adverse events, more severe pain) with prolonged daily oxygen use. All outcome variables are normalized to 11-point scales for ease of comparison. Adjusted for age, sex, BMI, underlying disease, blood gas analyses and smoking history. Estimates not crossing the dotted line (0) are interpreted as statistically significant. BMI: Body mass index, CAT: COPD assessment test.

In the linear regression analysis, longer reported daily oxygen use duration was significantly associated with higher total numbers of experienced adverse events, higher ESAS-r dyspnea and anxiety ratings, and higher total CAT scores in both crude and adjusted models (Table 3). Additional associations were seen in crude models for ESAS-r items depression and overall well-being, as well as for ESAS-r item pain in the adjusted analysis. No associations were seen for sleep quality or other ESAS-r items. A summary of regression estimates is presented in Figure 2.

**Table 3.** Associations between daily oxygen usage time, adverse events, symptoms, and health status.

	Mean shift in outcome variable per additional hour of daily oxygen duration	
	Crude estimate (95% CI)	Adjusted estimate (95% CI)
Total number of experienced adverse effects	0.14 (0.078–0.20)	0.16 (0.088–0.23)
Symptom severities		
Pain	0.044 (−0.028–0.12)	0.091 (0.0080–0.17)
Tiredness	0.60 (−0.0075–0.13)	0.043 (−0.041–0.13)
Drowsiness	0.056 (−0.014–0.13)	0.044 (−0.043–0.13)
Nausea	0.012 (−0.037–0.061)	0.054 (−0.013–0.12)
Lack of appetite	0.028 (−0.045–0.10)	0.062 (−0.030–0.15)
Dyspnea	0.11 (0.040–0.18)	0.12 (0.033–0.21)
Depression	0.10 (0.025–0.18)	0.089 (−0.0056–0.18)
Anxiety	0.11 (0.034–0.19)	0.11 (0.018–0.21)
Overall well-being	0.075 (0.0081–0.14)	0.077 (−0.006–0.16)
Worse sleep quality	−0.018 (−0.070–0.033)	−0.019 (−0.088–0.050)
CAT Score	0.078 (0.035–0.12)	0.093 (0.41–0.15)

Estimates using linear regression. Coefficients represent the mean shift in outcome variable per additional 1 hour of daily oxygen duration. A positive estimate thus reflects an increase in the outcome variable (i.e. higher number of adverse events, more severe pain) with prolonged daily oxygen use. All outcome variables are normalized to 11-point scales for ease of comparison. Estimates crude and adjusted for potential confounding factors (age, sex, BMI, underlying disease, blood gas analyses and smoking history). Bold estimates denote statistical significance.

CAT: Chronic obstructive pulmonary disease assessment test, CI: Confidence interval, ESAS-r: Revised Edmonton symptom assessment system.

## Discussion

### Main findings

In this population-based study of patients on LTOT, patients who used oxygen for more hours per day had a higher prevalence of adverse events, had higher symptom severity ratings, and reported worse health status, but reported a similar sleep quality and duration compared to patients with shorter daily oxygen use.

These findings complement earlier studies in several ways. While previous research has shown that adverse events and symptoms are common among patients treated with LTOT, no study has investigated the relationship between daily oxygen use duration and these outcomes.^20^ Reports of worse health status among patients reporting longer daily oxygen use in this study also complement and contrast the more limited data provided by the REDOX trial,^7^ where patients randomized to both 15 and 24 h of daily oxygen usage reported similar health status. While it is important to note that any causality in this association remains unclear, we hypothesize that an increased oxygen use duration may primarily be a response to progressive worsening of the underlying disease and/or symptoms, a theory supported by findings of a higher average amount of elapsed months on treatment among patients with a longer daily oxygen use duration. While this study can thus not be said to imply that a longer daily oxygen use duration causes a worsening of symptoms or health status outcomes, our findings would indicate that increasing the daily duration of oxygen therapy does not successfully normalize a worsening of these outcomes occurring naturally in disease progression. The associations found between a longer daily duration of oxygen use, higher numbers of local adverse events, and worse health status, could also reflect a cumulative impact of airway dryness and irritation with increased oxygen use, which is indirectly supported by previous findings of improved HrQoL with use of humidified high-flow oxygen.^29^

### Strengths and limitations

The primary strength of this study is the population-based sample of patients available from the national Swedevox registry, which also enabled comparison of study respondents and non-respondents using data from treatment baseline. This study also used previously validated instruments for measuring health status and symptom severity, and assessed adverse events based on previous literature and clinical experience.^30^

Limitations of this study include firstly the observational, cross-sectional design which does not allow for conclusions regarding causality, where longer daily oxygen use duration may be either a cause of, or a response to, worsening symptoms and health status. Secondly, the low survey response rate may increase the risk of selection bias due to potentially poorer health status among survey non-respondents, or better treatment compliance among respondents. While baseline characteristics were similar between respondents and non-respondents, no registry data was available from the time of survey completion. The self-reported source of data regarding primarily daily oxygen usage time may also be unreliable, due to shame or fear among respondents. Sleep duration and quality was measured using ordinal scales and not with previously validated sleep quality instruments, which were judged as infeasible in a postal survey directed to patients with severe illness.

### Implications for clinical practice and future research

Prescribing LTOT for 24 rather than 15 h per day has not been shown to reduce the risk of mortality or hospitalization for patients, but the effect of daily oxygen use time on patient-reported outcomes is less well studied. While notably limited by its observational design, this study does *not* provide evidence that better patient-reported outcomes are seen with a longer daily duration of oxygen use, and does thus *not* support prescription of longer daily oxygen use with the goal of improving patient well-being. Additionally, this study provides evidence that adverse events, daytime symptoms, and worse health status may be more common or severe among patients with a longer daily oxygen duration, irrespective of any causality, and that extra care may be warranted in the assessment of such patients.

For future research, we suggest the continuous incorporation of patient-report outcomes in randomized trials of LTOT.

## Supplemental Material

Supplemental Material - Longer daily oxygen use associates with more adverse events, symptoms, and worse health status in long-term oxygen therapy Supplemental Material for Longer daily oxygen use associates with more adverse events, symptoms, and worse health status in long-term oxygen therapy by Filip Björklund, Magnus Ekström in Chronic Respiratory Disease
