# Supplementary material for: Longer daily oxygen use associates with more adverse events, symptoms, and worse health status in long-term oxygen therapy
Source: Chron Respir Dis. 2025 Aug 8;22:14799731251366962. doi: 10.1177/14799731251366962 (PMC12334815; doi:10.1177/14799731251366962)

SUPPLEMENTAL MATERIAL

Supplemental table 1. Swedevox baseline characteristics of survey respondents and non-respondents.

	**Respondents** N=204	**Non-respondents** N=546
**Age at survey (years), mean (SD)**	75.3 (8.7)	74.9 (10.3)
**Female**	123 (60.3%)	374 (68.5%)
**Main underlying cause for LTOT**		
COPD	153 (75.0%)	371 (67.9%)
ILD	23 (11.3%)	58 (10.6%)
Other	27 (13.2%)	111 (20.3%)
Missing	1 (0.5%)	6 (1.1%)
**Baseline PaO_2 on air, kPa (mean, SD)**	6.6 (0.76)	6.5 (0.89)
**Baseline PaO_2 on oxygen, kPa (mean, SD)**	8.7 (1.1)	8.6 (1.3)
**Baseline $PaCO_2$ on air, kPa (mean, SD)**	5.8 (1.3)	5.9 (1.2)
**Baseline $PaCO_2$ on oxygen, kPa (mean, SD)**	5.9 (1.1)	6.1 (1.3)
**Baseline FEV1, L (median, IQR)**	1.1 (0.7-1.5)	0.9 (0.6-1.4)
**Baseline FEV1, % predicted (median, IQR)**	42.5 (31.5-58.0)	41.0 (28.4-62.0)
**WHO Performance status (mean, SD)**		
0	23 (11.3%)	61 (11.2%)
1	92 (45.1%)	201 (36.8%)
2	40 (19.6%)	115 (21.1%)
3	16 (7.8%)	47 (8.6%)

Abbreviations: COPD = Chronic obstructive pulmonary disease, FEV1 = Forced expiratory volume in one second, ILD = Interstitial lung disease, IQR = Interquartile range, LTOT = Long-term oxygen therapy, $PaCO_2$ = Partial pressure of carbon dioxide in arterial blood, PaO_2 = Partial pressure of oxygen in arterial blood, SD = Standard deviation, WHO = World Health Organization

Supplemental figure S1. Box plots displaying the relationship between daily oxygen use duration and sleep quality rated on a 5-point ordinal scale (1=worst, 5=best).

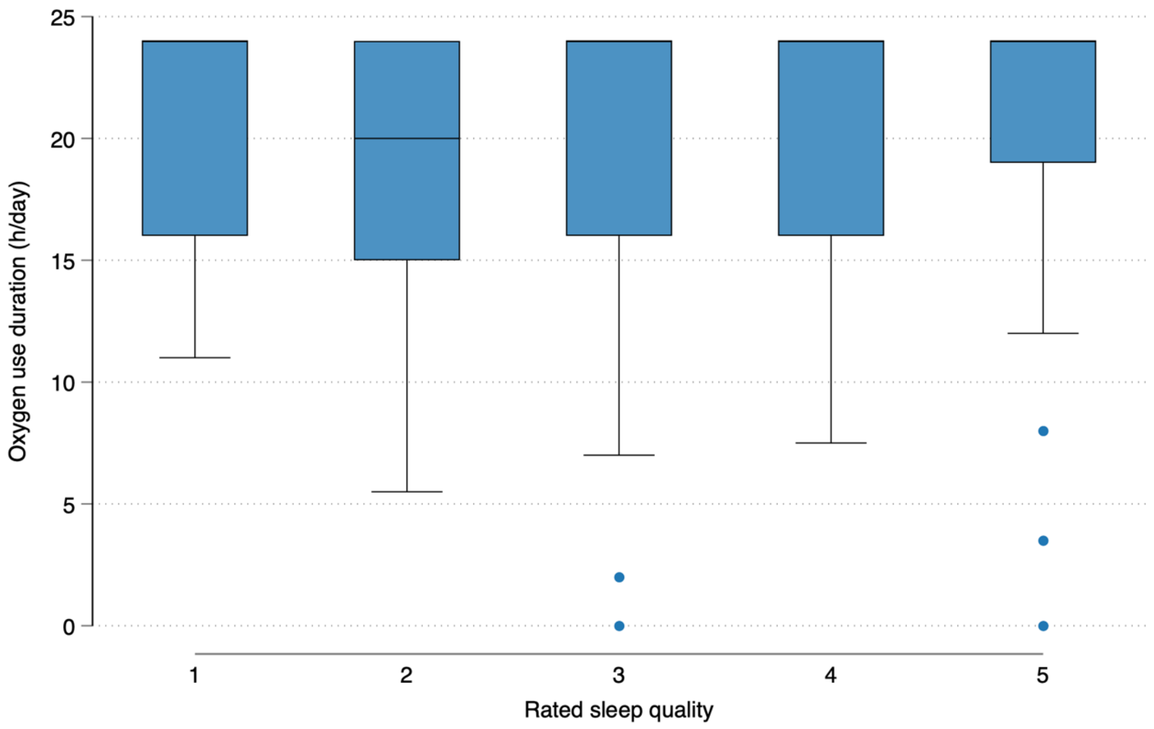

Supplemental figure S2. Jittered scatterplot with fitted trend line displaying the relationship between daily oxygen use time and total CAT score.

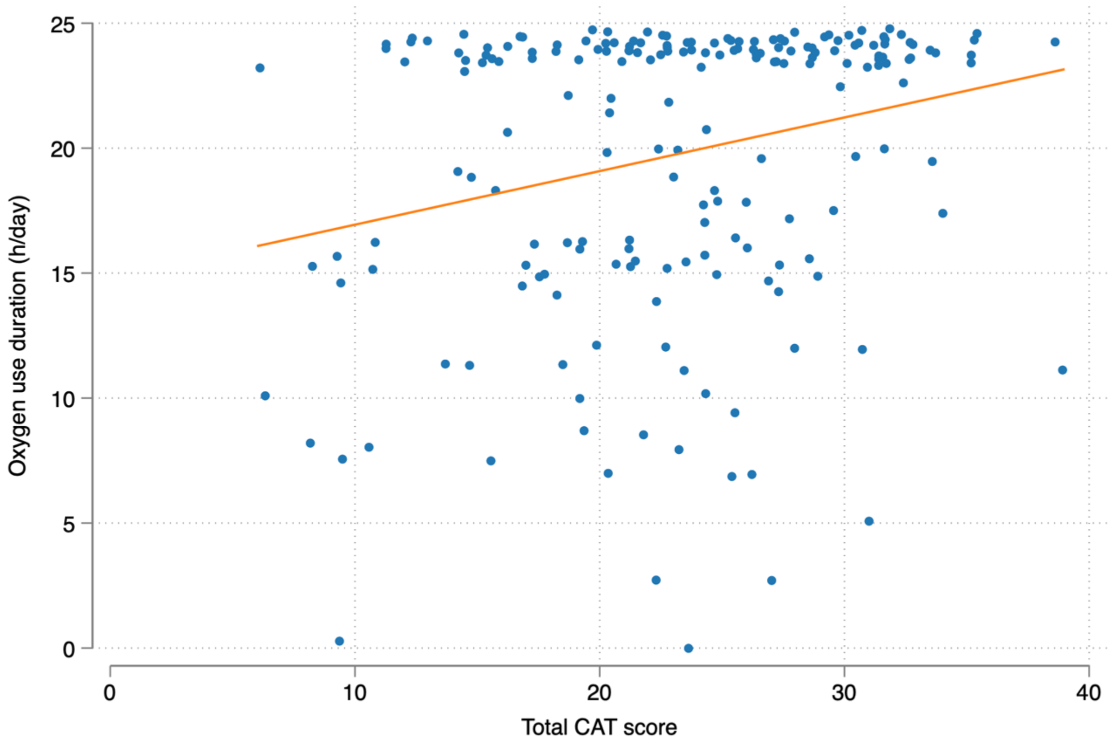

Abbreviations: CAT = Chronic obstructive pulmonary disease assessment test

Supplemental figure S3. Bar charts displaying the relationship between daily oxygen use time and symptoms rated using the ESAS-r.

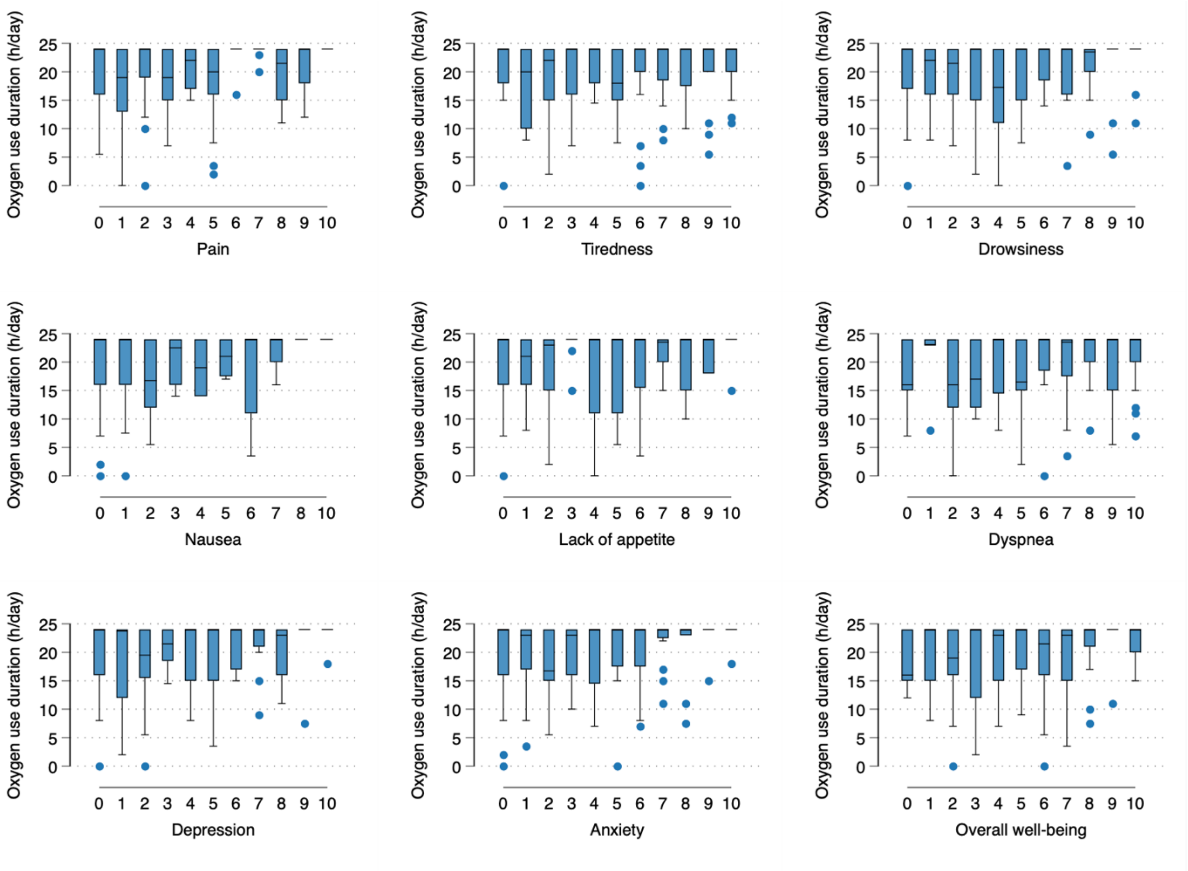

Supplement: Supplemental Material - Longer daily oxygen use associates with more adverse events, symptoms, and worse health status in long-term oxygen therapy [file sj-pdf-1-crd-10.1177_14799731251366962.pdf]
